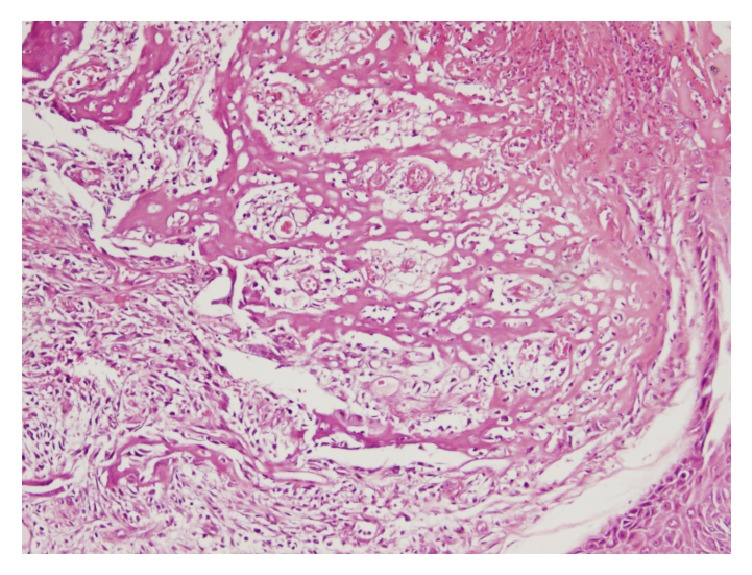# A Rare Extraskeletal Osteosarcoma Appearing After 55 Years on a Large Stage 3 Burn Scar

**DOI:** 10.1155/2018/5185604

**Published:** 2018-06-04

**Authors:** O. Vanhooteghem, I. Theate

**Affiliations:** ^1^Department of Dermatology, CHU UCL Sainte Elisabeth Hospital, Namur, Belgium; ^2^Department of Anatomopathology, IPG, Gosselies, Belgium

## Abstract

It is widely accepted that chronic burn wounds may lead to the development of various malignant skin tumors. Deep stage 3 burned areas may facilitate deeper carcinogenesis. Deep tissues are probably less subject to severe insult than is the epithelial layer during physical insult, suggesting that soft tissues transform to a lesser extent during the late stages of tumoral development as in an immunocompromised district with altered local immune defense with both cellular and humoral defense affected. Most authors claim that tumors are almost squamous cell carcinomas, although other types of malignancies such as basal cell carcinoma and, to a lesser extent, melanoma can also be seen. However, malignant transformation of cutaneous soft tissue in a burn insult area has rarely been described. Similarly, burn-induced tumors of histiocytic origin have been reported in few cases and osteosarcoma only in two case reports. Here, we report a patient case suffering from severe large stage 3 burn after-effects on the leg. Fifty-five years after the injury, this patient developed a large extraosseous osteosarcoma on the scar.

## 1. Case Report

**Figure 1:**
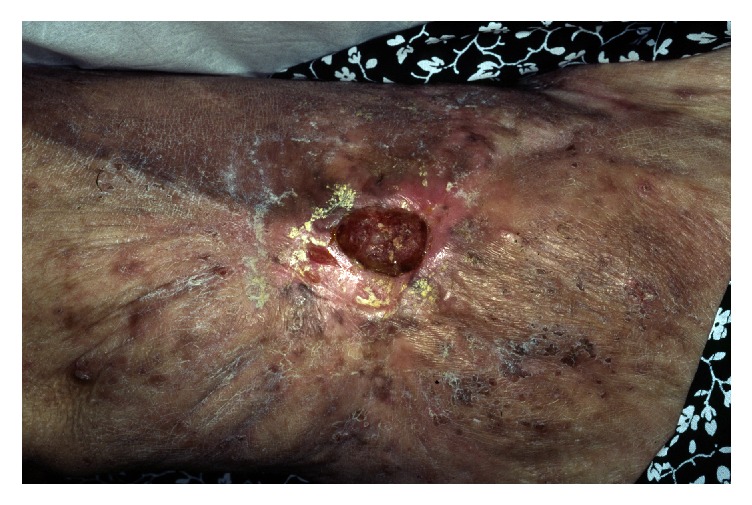


**Figure 2:**
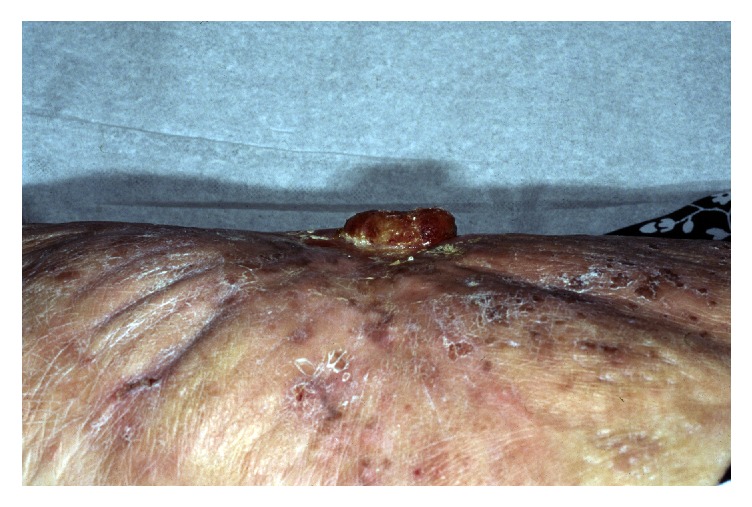


**Figure 3:**
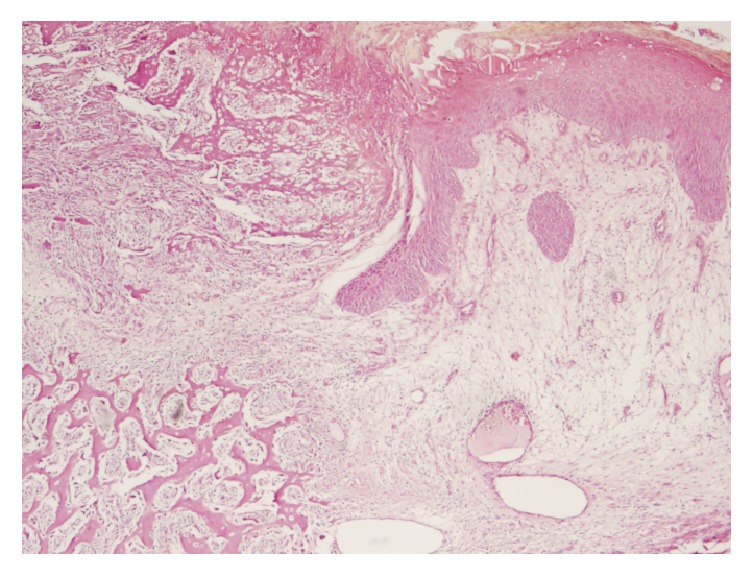


A 66-year-old phototype 6 woman from North-Africa was admitted to our department due to an indolent ulcerovegetative mass that developed on the anterior face of the right thigh. The lesion spontaneously exhibited greyish discharge. The patient anamnesis revealed that she had sustained a hot-water burn to her leg at the age of 11 years. She stated no definitive treatment by skin grafting and had only been treated conservatively following injury. The burned right thigh healed progressively, leaving a large scar on the skin surface. There had been no particular issues concerning the lesion until 2 months previously, at which point the patient noticed a progressive mass in the scar area but had not taken any steps to have it cured. Upon physical examination, the mass was 4 x 2 cm and suggested at diagnosis of Marjolin's ulcer (Figures 1 and 2). Histopathologic features of the surgical specimen were characterized by predominant chondroid matrix with marked cellularity, high grade atypia, and high mitotic activity. Foci of bone and osteoid formation were also seen (Figures 3 and 4). A diagnosis of cutaneous extraosseous osteosarcoma was suggested after excluding an origin in bone or other primary tumor sites by computed tomography. Indeed, clinical examination and extensive total body radiologic workup revealed absence of bone lesions in any body site. Unfortunately, the patient rejected the therapeutic proposal of definitive large surgical excision and we failed to obtain any notice regarding her further evolution.

## 2. Discussion

In addition to the aesthetic disturbance and functional inconvenience caused by large burn scars, these lesions facilitate the development of skin malignancy. According to the current literature, the risk of malignancy transformation reaches 2%, while the mean latency interval between onset of original insult and secondary tumoral occurrence is approximately 30 years [1–3] except in a patient who developed a squamous cell carcinoma 6 weeks after burning [4] and another in the year after the burn [5]. Among the skin cancers that develop on old burn scars, including predominantly the body extremities, squamous cell carcinoma (70%) is the most common, followed in incidence by basal cell carcinoma (12%), while other sarcomas develop only rarely [1, 6]. Basically, the pathophysiological mechanisms leading to malignant transformation of burn scars are not fully understood. However, the lag period prior to induction of malignancy is inversely proportional to patient's age at the time of the burn injury. Thus, younger patients tend to develop cancer after a much longer period of time, as has been the case in our experience. However, there is also a gender effect. Indeed, the risk cancer for burned female was significant and increased than burned male [7]

Extraskeletal osteosarcoma (EO) located in the soft tissues without attachment to the bone or periosteum is a malignant mesenchymal neoplasm that produces osteoid, bone, and /or chondroid material. EO occurs rarely with an incidence up to 4 to 5% of osteosarcoma and 1% of soft tissue sarcoma [8]. It affects adults almost exclusively with a high incidence in patients older than 50 years, more common in male patients. The tumor occurred principally in an extremity, with a predilection for the thighs. Up to 13% of cases have been reported with a history of prior trauma to the site of the tumor and/or radiotherapy [9]. To our knowledge, two cases of EO are described on a burned site [10, 11].

The best prophylaxis for the development of malignancy in chronic burn scars is to achieve a stable covering of the burn wound, either by skin graft or flap coverage [6]. However, the graft may carry a potential for malignant transformation. Melanoma can be transferred to the recipient site with the skin graft [12]. However, the burn scars are still out of immune control with poor perfusion and lymphatic drainage. This condition may facilitate deeper carcinogenesis, and deep tissues are probably less subject to severe insult than is the epithelial layer during physical insult, suggesting that soft tissues transform to a lesser extent during the late stages of tumoral development as in a immunocompromised district [13–15] with altered local immune defense with both cellular and humoral defense affected [16, 17].

In conclusion, in case of change of appearance of the graft or the scar, patients should be reported to their physicians early [4] or even well 46 years after a skin graft placement [18]. The physicians should pay greater attention to patients who exhibit burn healing by secondary intention, to wounds that are not healing appropriately, and to fragile burn scars that ulcerate easily as in our experience 55 years after a spontaneous healing wound. Physicians should also pay attention to burned patients who received biological treatment and those under immunosuppressive medication for graft treatment.

## Figures and Tables

**Figure 4:**